# Hydroxyurea-induced melanonychia

**DOI:** 10.1016/j.jdcr.2023.09.027

**Published:** 2023-10-14

**Authors:** Michael G. Buontempo, Zaima S. Chaudhry, Ruchi S. Raval, Kelly Mourtzanakis, Vignesh Ramachandran, Jerry Shapiro, Kristen Lo Sicco

**Affiliations:** a Ronald O. Perelman Department of Dermatology, New York University Grossman School of Medicine, New York, New York; b Department of Dermatology, Hackensack Meridian School of Medicine, Nutley, New Jersey; c Department of Dermatology, Mayo Hospital, King Edward Medical University, Lahore, Punjab, Pakistan

**Keywords:** drug-induced side effects, hydroxyurea, hyperpigmentation, management of hydroxyurea-induced melanonychia, melanoma, melanonychia, nail biopsy, nail changes, thrombocytosis, transverse melanonychia

## Introduction

“Melanonychia” describes a brown/brown-black pigmented longitudinal or transverse band on the fingernail/toenail in response to reactive and neoplastic disorders and as a side effect of chemotherapeutic and systemic treatments.^1^ Hydroxyurea (HU), a cytostatic drug used in managing myeloproliferative disorders, can cause mucocutaneous adverse reactions, including hyperpigmentation, lichenoid eruptions, alopecia, and leg ulcers. However, the concurrent appearance of transverse melanonychia with homogenous linear hyperpigmentation bands during long-term HU therapy is relatively rare, with only 6 reported cases to date. This presentation can pose a diagnostic challenge as the differential diagnosis includes subungual melanoma, pigmented squamous cell carcinoma, subungual hematoma, nevus, and conditions related to ethnic predisposition and hyperpigmentation induced by other drugs.^2^

This report documents the case of a 61-year-old woman who developed linear homogenous hyperpigmentation and concomitant transverse melanonychia 6 months after initiating HU for thrombocytosis.

## Case report

A 61-year-old African American woman without personal or family history of cutaneous malignancies presented to our dermatology clinic. Her notable medical history includes peptic ulcer disease and long-standing thrombocytosis. Her thrombocytosis was detected 8 months prior to her presentation at our clinic, although the exact duration remains undetermined.

Six months prior to presenting to us, she began HU as a treatment for her thrombocytosis, which initially manifested with a platelet count exceeding 900,000 platelets/μL but has since reduced to approximately 400,000 platelets/μL. The patient reported good tolerance of the medication, except for the development of nail darkening at the eponychium and the emergence of linear nail stripes approximately 3 months after initiation of the HU. She reported no associated pain or tenderness.

Regarding lifestyle habits, the patient reported significant engagement in typing activities. She denied any occupational chemical or dye exposure, overt episodes of physical trauma to her fingers or toes, or a history of nail changes or onychomycosis.

**Fig 1:**
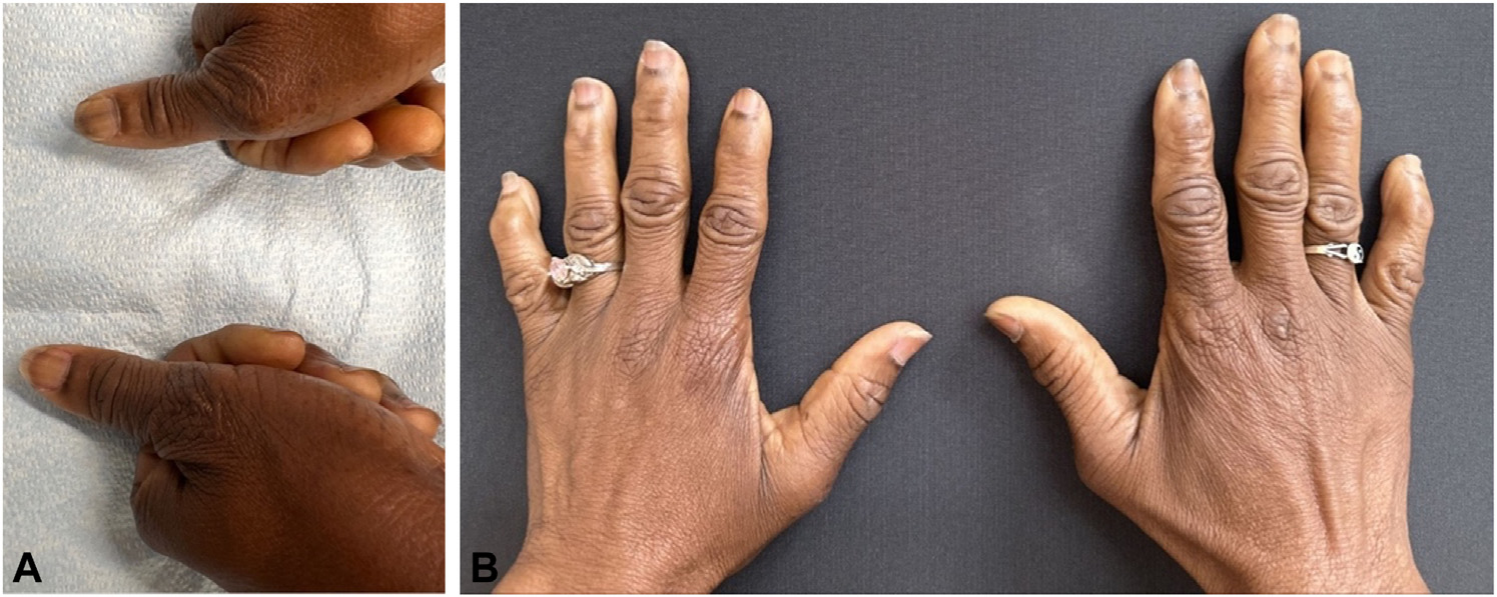
Hands. **A,** A detailed view of the patient’s thumb’s ungueal unit, highlighting the presence of linear melanonychia. The hyperpigmented bands are observed as well-defined, longitudinal streaks coursing from the proximal nailfold to the distal free edge of the nail plate. Their width measures less than 3 mm, aligning with the clinical presentation of melanonychia induced by hydroxyurea therapy. **B,** A panoramic view of the patient’s digital ungueal units in both hands. It is evident from the presentation that multiple fingernails manifest hyperpigmentation starting at the proximal nailfold and extending longitudinally across the nail plate. These linear bands are present uniformly across all digits, accentuating the distinctiveness of this manifestation. The ubiquity and consistency of the hyperpigmentation in all the nails underscore a potential systemic etiology, corroborated by the patient’s hydroxyurea therapy.

**Fig 2:**
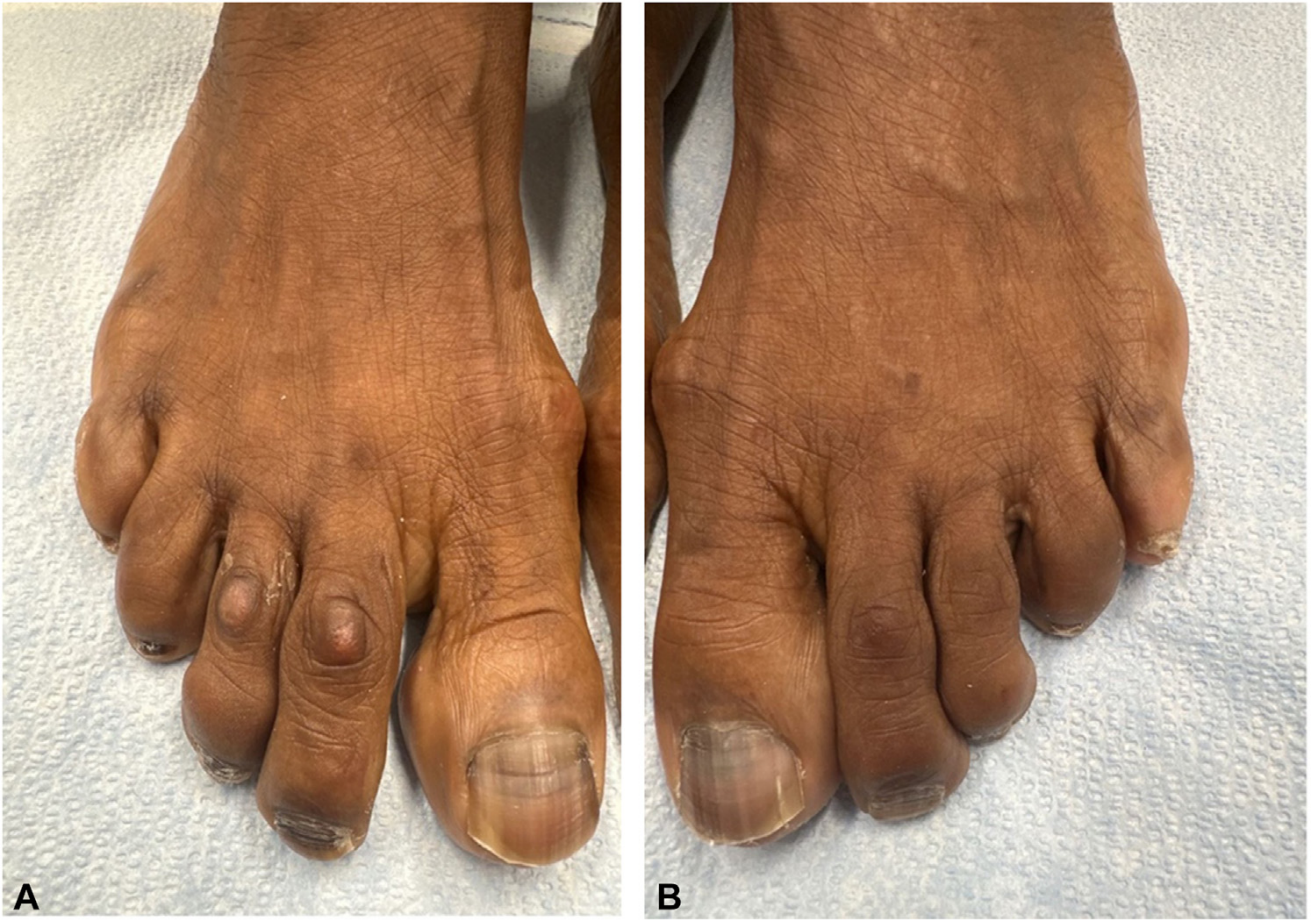
Feet. **A,** The patient’s right pedal ungueal unit revealing transverse bands of melanonychia. Notably, the degree of hyperpigmentation is striking and not restricted to the nail plate but extending to encompass the periungueal tissues, thereby highlighting the systemic origin of the pigmentation. **B,** The patient’s left pedal ungueal unit depicting a consistent pattern of transverse melanonychia as evidenced in the preceding images. The bilaterally symmetrical manifestation across both feet emphasizes the systemic implications of hydroxyurea therapy and differentiates it from localized etiologies of ungueal hyperpigmentation. Such consistent patterns observed bilaterally on both the manus and pes emphasize the widespread influence of hydroxyurea therapy on ungueal pigmentation.

On physical examination, we observed hyperpigmentation at the proximal nailfolds of multiple fingernails and toenails (Figs 1, *A* and *B*, and 2, *A* and *B*). Additionally, we identified linear hyperpigmented bands on her fingernails, all exhibiting a homogenous gray appearance via dermatoscopy and measuring less than 3 mm in width (Fig 1, *A*).

## Discussion

HU has been associated with a variety of cutaneous adverse reactions, notably mucocutaneous hyperpigmentation, affecting between 10% and 35% of patients on chronic HU therapy. Additional side effects include oral ulcers, stomatitis, oropharyngeal pigmentation, nonscarring alopecia, facial erythema, actinic keratosis–like lesions, and nonmelanoma skin cancers.^3^

A notable and significant observation in the development of melanonychia is the variation in reports of incidence, ranging from 4.3% to 55% of patients receiving HU therapy.^4^^,^^5^ Additionally, the reported onset of melanonychia after initiation of HU also varies, with a reported mean onset varying from 6.4 weeks to 11 months after the initiation of therapy.^4^^,^^5^

Although exact mechanisms underlying HU-induced melanonychia remain unclear, the literature suggests a likely multifactorial cellular and molecular approach. HU inhibits ribonucleotide reductase, causing cell cycle arrest and biochemical changes in redox and iron metabolism.^6^ Changes in iron metabolism may contribute to increased pericellular iron content, which further promotes melanocyte activation and melanin synthesis.^7^ The capillary-rich nail apparatus may also serve as a favorable environment for HU accumulation, especially in the lower extremities where there is stasis of blood flow. In the case of our patient, for example, repetitive typing as well as chronic walking with tight footwear, especially in the setting of potential anatomical variations, may have also contributed toward melanocyte activation. Together, these complex mechanisms facilitate increased melanin production in the nail matrix.

We conducted a PubMed search using the term “hydroxyurea induced melanonychia” OR “hydroxyurea” AND “melanonychia,” yielding 33 case studies. With a focus on HU-induced melanonychia, we categorized the results on the basis of the indication for HU: platelet disorders, anemia, cancer, HIV, and stroke. Among the results, 1 in 3 cases were related to HU given for the treatment of platelet disorders, relevant to our case of a 61-year-old female patient with a history of thrombocytosis (Supplementary Table I, available via Mendeley at https://doi.org/10.17632/g5drx8ndys.1).

Melanonychia typically presents as longitudinal bands on the nail unit and is more prevalent in individuals with darker-pigmented skin.^1^ The diagnosis of melanonychia necessitates a detailed patient history, including the onset, duration, associated symptoms, changes in the coloration of the nail unit or surrounding skin, and the size and uniformity of the bands.^1^ Additionally, a physical examination should note the number of nails involved, the location and direction of the discoloration, and the presence of nail dystrophy.^1^

Nail changes due to antineoplastic drugs are generally asymptomatic and reversible within a few months after drug discontinuation. However, 1 case report described recurrent melanonychia upon reinitiation of HU.^8^ This lack of outcome data highlights a gap in the literature and an opportunity to investigate various treatment strategies to manage hyperpigmentation without interrupting the HU treatment regimen.

Differentiating melanonychia caused by HU from more serious conditions such as subungual melanoma is crucial. Subungual melanoma or pigmented squamous cell carcinoma may present similarly, especially in non-Caucasian populations, and might necessitate a nail matrix biopsy, potentially leading to permanent nail deformity. However, melanonychia in the setting of melanocyte activation typically occurs in multiple nails, whereas subungual melanoma may occur in a single nail unit. The presence of Hutchinson’s sign and micro-Hutchinson’s sign is a key clinical indicator of subungual melanoma.^9^^,^^10^

Although discontinuing HU often results in improvement or complete resolution of nail hyperpigmentation, not all patients are able to discontinue treatment. This underlines the need for further exploration of alternative strategies to manage this side effect while maintaining the HU regimen.

To that end, topical applications such as urea cream, which functions as a keratolytic, can potentially assist in lightening pigmentation. Further research is required to establish its effectiveness and suitability herein.

In conclusion, HU-induced melanonychia is a clinical phenomenon warranting a comprehensive understanding of its presentation, diagnosis, and management. This report and literature review aim to provide a holistic review of the current knowledge on this topic, contributing to the broader discourse on patient management in the context of HU therapy.

## Conflicts of interest

Dr Lo Sicco is a consultant for Pfizer and Aquis. Dr Shapiro is a consultant for Aclaris Therapeutics, Incyte, and Replicel Life Sciences. Drs Shapiro and Lo Sicco have been investigators for Regen Lab and are investigators for Pfizer. Authors Buontempo, Raval, and Mourtzanakis and Drs Chaudhry and Ramachandran have no conflicts to disclose.

## References

[1] Buka R., Friedman K.A., Phelps R.G., Silver L., Calero F., Rudikoff D. (2001). Childhood longitudinal melanonychia: case reports and review of the literature. Mt Sinai J Med.

[2] Lee D.K., Lipner S.R. (2022). Optimal diagnosis and management of common nail disorders. Ann Med.

[3] Kalajian A.H., Cely S.J., Malone J.C., Burruss J.B., Callen J.P. (2010). Hydroxyurea-associated dermatomyositis-like eruption demonstrating abnormal epidermal p53 expression: a potential premalignant manifestation of chronic hydroxyurea and UV radiation exposure. Arch Dermatol.

[4] Aste N., Fumo G., Contu F., Aste N., Biggio P. (2002). Nail pigmentation caused by hydroxyurea: report of 9 cases. J Am Acad Dermatol.

[5] Kumar B., Saraswat A., Kaur I. (2002). Mucocutaneous adverse effects of hydroxyurea: a prospective study of 30 psoriasis patients. Clin Exp Dermatol.

[6] Chitambar C.R., Wereley J.P. (1995). Effect of hydroxyurea on cellular iron metabolism in human leukemic CCRF-CEM Cells: changes in iron uptake and the regulation of transferrin receptor and ferritin gene expression following inhibition of DNA synthesis. Cancer Res.

[7] Lee K.P., Vangipuram R.K., Klimas N.K., Sanyal S., Koshelev M.V. (2019). Hydroxyurea-induced hyperpigmentation with iron deposition. Dermatol Online J.

[8] Sun Y., Xu Z., Ding X. (2022). Recurrent melanonychia on multiple nails and alopecia induced by hydroxyurea therapy. Chin Med J (Engl).

[9] Baran R., Kechijian P. (1996). Hutchinson’s sign: a reappraisal. J Am Acad Dermatol.

[10] Koga H., Saida T., Uhara H. (2011). Key point in dermoscopic differentiation between early nail apparatus melanoma and benign longitudinal melanonychia. J Dermatol.

